# Localized anesthesia of a specific brain region using ultrasound-responsive barbiturate nanodroplets

**DOI:** 10.7150/thno.41566

**Published:** 2020-02-03

**Authors:** Harriet Lea-Banks, Meaghan A. O'Reilly, Clement Hamani, Kullervo Hynynen

**Affiliations:** 1 Physical Sciences Platform, Sunnybrook Research Institute, Toronto, Canada; 2 Department of Medical Biophysics, University of Toronto, Toronto, Canada; 3 Division of Neurosurgery, Sunnybrook Health Sciences Centre, University of Toronto, Toronto, Canada; 4 Harquail Centre for Neuromodulation, Sunnybrook Research Institute, Sunnybrook Health Sciences Centre, University of Toronto, Toronto, Canada; 5 Institute of Biomaterials and Biomedical Engineering, University of Toronto, Toronto, Canada

**Keywords:** focused ultrasound, triggered drug delivery, phase-change emulsion

## Abstract

**Background**: Targeted neuromodulation is a valuable technique for the study and treatment of the brain. Using focused ultrasound to target the local delivery of anesthetics in the brain offers a safe and reproducible option for suppressing neuronal activity.

**Objective**: To develop a potential new tool for localized neuromodulation through the triggered release of pentobarbital from ultrasound-responsive nanodroplets.

**Method**: The commercial microbubble contrast agent, Definity, was filled with decafluorobutane gas and loaded with a lipophilic anesthetic drug, before being condensed into liquid-filled nanodroplets of 210 ± 80 nm. Focused ultrasound at 0.58 MHz was found to convert nanodroplets into microbubbles, simultaneously releasing the drug and inducing local anesthesia in the motor cortex of rats (n=8).

**Results**: Behavioral analysis indicated a 19.1 ± 13% motor deficit on the contralateral side of treated animals, assessed through the cylinder test and gait analysis, illustrating successful local anesthesia, without compromising the blood-brain barrier.

**Conclusion**: Pentobarbital-loaded decafluorobutane-core Definity-based nanodroplets are a potential agent for ultrasound-triggered and targeted neuromodulation.

## Introduction

Modulating neuronal activity in the brain is desirable in a variety of treatment and research applications, from suppressing swelling following traumatic brain injury 1, to mapping functional networks 2. Deep brain stimulation (DBS) is a popular technique for localized stimulation of cerebral tissue, and has found use in treating Parkinson's disease, essential tremor, epilepsy and OCD. However, DBS is invasive, requiring electrodes to be implanted into the cerebral tissue and, although infrequently, this technique carries a risk of side effects 3.

Focused ultrasound (FUS) offers a non-invasive alternative and has been shown to achieve transient neurostimulation in a safe and targeted manner 4. Combining FUS with a pharmaceutical agent can modulate neural activity with spatial and temporal specificity. Recently this has been shown through the direct delivery of the neurotransmitter gamma-Aminobutyric acid (GABA) across the open blood-brain barrier (BBB), following MRI-guided FUS treatment 5. Since GABA is not able to cross the intact BBB, targeting with FUS in the presence of microbubbles allows a specific brain region to become permeable to the neurotransmitter.

However, many small molecule agents do not require the BBB to be compromised. Recently the delivery of propofol was achieved using ultrasound-responsive nanocarriers, known as nanodroplets, loaded with the anesthetic 2,6. Nanodroplets are sub-micron liquid-filled particles that vaporize into gas-filled microbubbles when exposed to ultrasound. Using ultrasound-responsive nanodroplets as drug-carriers has several benefits (as reviewed by Lea-Banks *et al.* 7). Firstly, drug encapsulation prevents systemic delivery and off-target effects 8. Secondly, nanodroplets exhibit a significantly longer circulation time than microbubble formulations 9, attesting their stability and extending the potential treatment window. Finally, since unique acoustic signals are generated during nanodroplet activation 10, drug delivery may be monitored through the detection of ultrasound emissions.

Previous studies exploring the local delivery of an anesthetic have implemented novel polymer nanodroplets filled with a high-boiling-point perfluorocarbon 2,6, requiring significant ultrasound pressures for vaporization and the use of experimental materials. Building on the important body of work using volatile perfluorocarbons to reduce the vaporization threshold of nanodroplets 11**,** 12, here we show for the first time how Definity, a clinically approved ultrasound contrast agent, may be repurposed as a nano-scale drug-carrier for localized neuromodulation. By using a low-boiling-point perfluorocarbon core - decafluorobutane - inside an agent formed from clinically approved components, we were able to achieve a 33 - 56 % reduction in vaporization threshold compared to perfluoropentane with a polymer shell where 1.2 - 1.8 MPa was necessary, with 100 ms pulse length rather than 10 ms used in the current study 2,6. Pentobarbital-loaded decafluorobutane-core Definity-based nanodroplets were sonicated at the motor cortex and induced local anesthesia, detected through behavioral changes and a contralateral motor deficit.

## Methods

### Drug-loaded nanodroplets

#### Materials

Decafluorobutane (C_4_F_10_) was purchased from Synquest Labs, US; pentobarbital sodium, under the brand name Euthanyl, from CDMV Inc., Quebec; and Definity from Lantheus Medical Imaging. Milli-Q ultrapure water (MilliporeSigma) was used throughout the fabrication.

Definity microbubbles are a commercially available FDA-approved ultrasound contrast agent. An octafluoropropane (C_3_F_8_) core is encapsulated by a lipid shell composed of (R)-4-hydroxy-N,N,N-trimethyl-10-oxo-7-[(1oxohexadecyl)oxy]-3,4,9-trioxa-4-phosphapentacosan-1-aminium, 4-oxide, (DPPC); (R)-hexadecanoic acid, 1-[(phosphonoxy)methyl]-1,2-ethanediyl ester (DPPA); and (R)-∝-[6-hydroxy-6-oxido-9-[(1-oxohexadecyl)oxy]-5,7,11-trioxa-2aza-6-phosphahexacos-1-yl]-ω-methoxypoly(ox-1,2-ethanediyl) (MPEG5000 DPPE), at a weight ratio of 82:10:8, giving a lipid concentration of 0.75 mg/mL. Manufacturer specifications state 1.2x10^10^ microbubbles/ mL are formed by agitation, with a mean diameter range of 1.1 - 3.3 μm.

#### Fabrication

Pentobarbital-loaded nanodroplets were formed through the modification of the clinically-approved ultrasound contrast agent, Definity. The fabrication protocol was based on a previously reported condensation technique 13, with the addition of 200 μL of 1 mg/mL sodium pentobarbital (Euthanyl) added to a vial of Definity (1.6 mL of 0.75 mg/mL lipid solution). A tip sonicator (S-450D, Branson Ultrasonics, USA) was used to disrupt the lipid membrane and integrate the lipophilic component of the drug (pentobarbital), using a 3 mm tip at 10% power, 20 s of sonication pulsed at 1 s on, 1 s off. The vial was sealed and connected to a vacuum pump where the original gas (octafluoropropane) was removed and replaced with decafluorobutane. The vial was agitated for 45 s using the VialMix(Lantheus Medical Imaging, USA) to form a white solution of precursor microbubbles. A bath of isopropanol was prepared with dry ice and cooled to -10°C. The vial was submerged and swirled gently for 2 min until the white solution became transparent, signaling successful condensation of microbubbles into nanodroplets.

Removing free drug and remaining microbubbles was achieved through centrifugation at 200 G for 5 min at 4°C, where the supernatant was replaced with cold Milli-Q ultrapure water and repeated 3 times. The pellet was gently re-suspended each time with several pumps of the pipette. Erroneous large droplets were removed in a final purification step by passing the droplet solution slowly through a 0.8 μm sterile syringe filter (Minisart Syringe Filter, Sartorius, Germany) into a tube on ice. The final solution was transparent and free of microbubbles. Nanodroplets were stored on ice and used within 4 hours of fabrication.

#### Characterization

Nanodroplet size distribution was assessed by nanoparticle tracking analysis (NanoSight, Malvern Panalytical, UK) for 3 independent batches, alongside zeta potential measurements (ZetaSizer, Malvern Panalytical, UK). Pentobarbital was quantified for drug-loading and release through absorbance measurements (NanoDrop™ 2000c Spectrophotometer, Thermo Scientific, USA) using a UV-compatible disposable cuvette. A standard curve was performed to quantify pentobarbital concentration through UV absorbance (Figure 3A).

It has been reported previously that DFB nanodroplets typically undergo an expansion ratio of 5.12 during vaporization 10. This was verified experimentally for our formulation by taking size measurements of the resultant microbubbles using a Coulter Counter (Multisizer 3, Beckman Coulter, USA) with 30μm aperture. Nanodroplets from 3 independent batches were allowed to spontaneously vaporize after several hours at room temperature in filtered phosphate-buffered saline solution (Beckman Coulter^TM^ ISOTON^TM^ II Diluent). Brightfield microscopy images (using the Zeiss Axio Imager M2 at 20x magnification) were also captured before and after vaporization to visualize the production of micron-scale bubbles.

The acoustic response of nanodroplets *in vitro* was measured using an in-house manufactured prototype system similar to the LP100 (FUS Instruments Inc., Toronto, ON, Canada) (further detail in the following section), with transducer and detector coaxially aligned to a 1.1 mm internal diameter tube. The aqueous suspension of nanodroplets was sonicated with 10 ms bursts at a flow rate of 0.2 mL/min. The effluent was collected and the released drug was extracted using an organic solvent sink of hexane and ethyl acetate (1:9 volume ratio), and transferred into ethanol for UV-vis measurements. The equivalent assessment was performed without FUS to assess spontaneous drug release and stability at 4°C, 23°C and 37°C over 5 hours.

### MRI-guided FUS

#### Animals

Male Sprague Dawley rats (n = 33) were purchased from Taconic Biosciences (Germantown, NY, USA) and had a mean weight of 394 ± 71 g on the day of experimentation. Rats were divided at random between 6 experimental groups (table 1). Animals were housed in the Sunnybrook Research Institute animal facility (Toronto, ON, Canada) on a reverse light cycle and had access to food and water *ad libitum*. All animal procedures were approved by the Animal Care Committee at Sunnybrook Research Institute and are in accordance with the Canadian Council on Animal Care and ARRIVE guidelines.

#### Animal preparation

General anesthesia was induced using 5% isoflurane (ISO), then maintained at 2% for the duration of the MRI-guided FUS procedure. Depilatory cream was used to remove hair from the scalp to avoid air bubbles and enable sufficient coupling with the ultrasound gel. A 22 gauge tail vein catheter was placed and the animal was positioned supine on top of the ultrasound system, breathing into a nose cone secured with a bite bar. A warm saline bag was placed on the animal torso to maintain body temperature during sonication.

#### MRI-guided FUS

**Figure 1:**
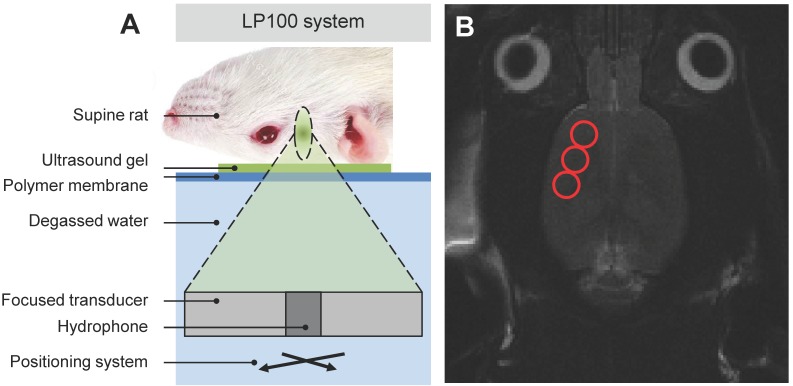
Schematic of **(A)** LP100 focused ultrasound system targeting the right motor cortex of a supine rat with **(B)** MRI guidance selecting three FUS targets.

Focused ultrasound (FUS) was delivered using the ultrasound system (described above) with a spherically focused transducer (580 kHz center frequency, 60 mm radius of curvature, 75 mm external diameter with circular cut-out of 20 mm diameter in the center of the transducer), calibrated using a planar fiber optic hydrophone with an active tip diameter of 10 μm (Precision Acoustics Ltd., Dorset, UK), coaxially aligned to a narrowband PZT hydrophone tuned at the subharmonic frequency with 20 mm diameter (Figure 1A). The system includes a tank of degassed and deionized water through which the transducer is moved using a motorized positioning system. The surface of the water is coupled to a polyimide membrane which in turn is coupled, using ultrasound gel, to the animal's skull. As in previous studies 14,15, the spatial coordinates of the LP100 system were co-registered with the 7-Tesla MRI scanner (BioSpec 70/30 USR, Bruker, Billerica, MA, USA), allowing FUS to be targeted using axial T2-weighted images. In the current study three focal spots were chosen to cover either the left or right side of the motor cortex (Figure 1B), where each ultrasound focus had a volume of 3 x 3 x 20 mm, located at approximate RAS coordinates (-3.0, 2.0, 3.0 mm), (-4.0, 0.0, 3.0 mm) and (-5.0, -2.0, 3.0 mm) respectively.

Drug-loaded nanodroplets were delivered via a 200 μL bolus injection immediately before FUS exposure, containing approximately 2x10^10^ nanodroplets and 5 μg of pentobarbital. This dosing scheme was based on studies showing impaired spatial learning and memory following direct micro-injection of pentobarbital (1.8 μg) into the hippocampus 16. We reasoned that a similar dose applied to motor cortical regions would lead to impaired mobility in the contralateral side of the body.

Nanodroplets were followed by a 250 μL saline flush, 0.2 mL/kg of gadolinium-based contrast agent (Gadovist, Schering AG, Berlin, Germany) and a final 250 μL flush. All injections were administered through the tail vein catheter. FUS was pulsed in 10 ms bursts at a repetition frequency of 1 Hz for a total duration of 180 s. A pressure ramp was used to detect the onset of droplet activation, starting at peak negative pressure of 0.32 MPa and increasing by 6.4 kPa each second until droplet vaporization was detected. 10 s of baseline measurements were recorded at 0.32 MPa prior to droplet injection. All *in vivo* pressure values are derated assuming 76% transmission through the rat skull at 580 kHz 17 and transmission through 5 mm of brain tissue, assuming an attenuation coefficient of 5 Np/m/MHz 18.

Droplet vaporization was detected using an in-house fabricated narrowband PZT hydrophone located in the central cut-out of the transducer. The control algorithm implemented was based on the magnitude of the ultraharmonic emissions, as previously used in monitoring safe blood-brain-barrier opening 19. Once the magnitude of ultraharmonic emissions exceeded 10 times the standard deviation of the mean baseline signal, the sonication pressure was fixed for the remainder of the treatment. T1- and T2*-weighted images were captured immediately following FUS exposure to assess gadolinium extravasation (indicating BBB opening) and red blood cell extravasation (indicating vessel damage).

#### Nanodroplet circulation and persistence

In a supplementary study, 10 animals were used to assess the acoustic persistence of the nanodroplets following a single 200 μL bolus injection (9 rats receiving sham droplets, 1 rat receiving pentobarbital-loaded droplets). The brain was sonicated at a fixed pressure of 0.8 MPa with 10 ms pulses, at four distinct central-brain locations for 5 s, repeated every minute over a 15 min total duration. Nanodroplet persistence was quantified by monitoring the decay in ultraharmonic emissions. The circulation half-life of sham and pentobarbital-loaded droplets was compared.

### Behavioral assessment

**Figure 2:**
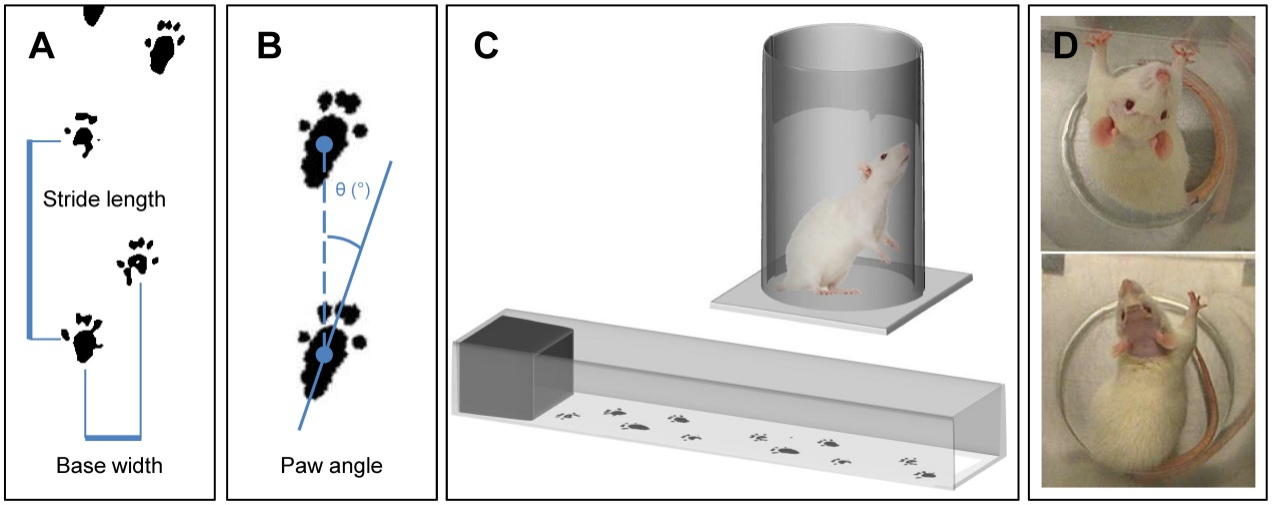
Behavioral testing using gait analysis measuring **(A)** stride length, base width and **(B)** paw angle. **(C)** Schematics of gait analysis and cylinder test apparatus, and **(D)** example images from the cylinder test showing simultaneous paw use pre-treatment and ipsilateral paw use post-treatment.

Two forms of behavioral testing were chosen based on reputable assessment of asymmetric motor function in the context of traumatic brain injury 20. Firstly, gait analysis was carried out to assess weakness in the contralateral hind limb. The hind paws of the rat were dipped in paint (Crayola Washable Fingerpaint) and the animal was allowed to walk along a strip of paper housed in a channel (1 m x 90 mm x 150 mm) with a dark enclosure at the far end (Figure 2C). The resultant footprints were assessed for stride length, base width and paw angle (Figure 2A, 2B). In particular, an increase in paw angle is associated with motor impairment 20.

Secondly, to assess ipsilateral fore limb preference, the cylinder test was used. A polymethyl methacrylate (PMMA) cylinder, 20 cm in diameter and 30 cm tall, was used with a camera suspended above (Figure 2C). Behavior was recorded for 5 min and the number of right and left forelimb touches to the internal wall of the cylinder were counted by a researcher blinded to the treatment groups (Figure 2D).

### Tissue assessment

Histology was performed on an example animal to assess tissue damage and red blood cell extravasation in the sonicated region, as well as systemic toxicity in other major organs - liver, lungs, kidney, spleen and heart. The animal was perfused with saline, then 10% formaldehyde in saline two hours following treatment with pentobarbital-loaded nanodroplets and FUS. The organs were transferred into 70% ethanol before being embedded in paraffin wax. 5 μm thick axial sections were taken at 500 μm separation, which then received haematoxylin and eosin (H&E) staining.

## Results

### Drug-loaded nanodroplets

#### Characterization

The lipophilic drug, pentobarbital, was loaded into the Definity-based lipid shell, as illustrated in Figure 4A. Average nanodroplet diameter was measured as 210 ± 80 nm from 3 independent batches (Figure 4B), with an average stock concentration of 1.03 x 10^11^ p/mL, measured at 1:10 dilution. The nanodroplets had a zeta potential of -8.7 ± 1.1 mV. Following vaporization, resultant microbubbles were found to have a mean diameter of 1.08 ± 0.06 μm (Figure 4C), giving an average expansion ratio of 5.14, in close agreement with that recorded by Sheeran *et al.* 10 using ultra-high-speed video measurements of vaporizing DFB nanodroplets who reported a 5.12 average expansion ratio.

#### Vaporization and ultrasound-mediated drug-release

**Figure 3:**
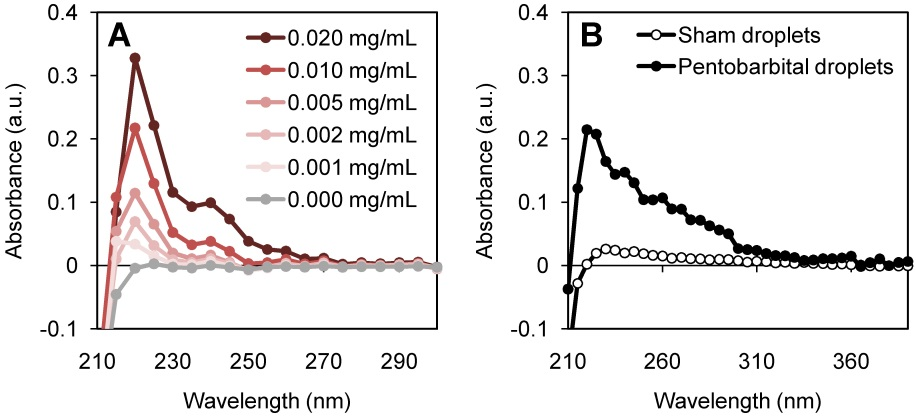
UV-vis measurements to quantify pentobarbital concentration for **(A)** standard curve and **(B)** final droplet solution measured at a 1:2 dilution. Pentobarbital dilutions were used to quantify pentobarbital loading, showing 20 - 24 % loading efficiency of pentobarbital into Definity-based lipid droplets, achieving 22 ± 3 μg/mL.

By sonicating the salt derivative, sodium pentobarbital (10 mg/mL, pH 9.5), in a dilute aqueous solution (1 mg/mL, pH 7) a significant amount of sodium (Na+) disassociated, leaving the hydrophobic pentobarbital. We hypothesize that the production of pentobarbital was enabled by the combination of the neutral pH environment and the violent cavitation events from the tip sonicator producing free radical species 21. This process was confirmed by sonicating an aqueous dilution of sodium pentobarbital (1 mg/mL) and using an organic solvent sink to extract the resultant hydrophobic drug. The produced pentobarbital was captured in hexane-ethyl acetate (1:9) and transferred into ethanol for UV-vis measurements (Figure 3A).

A lipid to drug weight ratio of 6:1 was found to be optimal for fabrication and loading. A higher proportion of lipophilic drug prevented precursor microbubbles from being formed; a lower proportion reduced the potential therapeutic effect. A loading efficiency of 20 - 24% was achieved, resulting in 22 ± 3 μg of pentobarbital in 1 mL of nanodroplets (Figure 3B).

**Figure 4:**
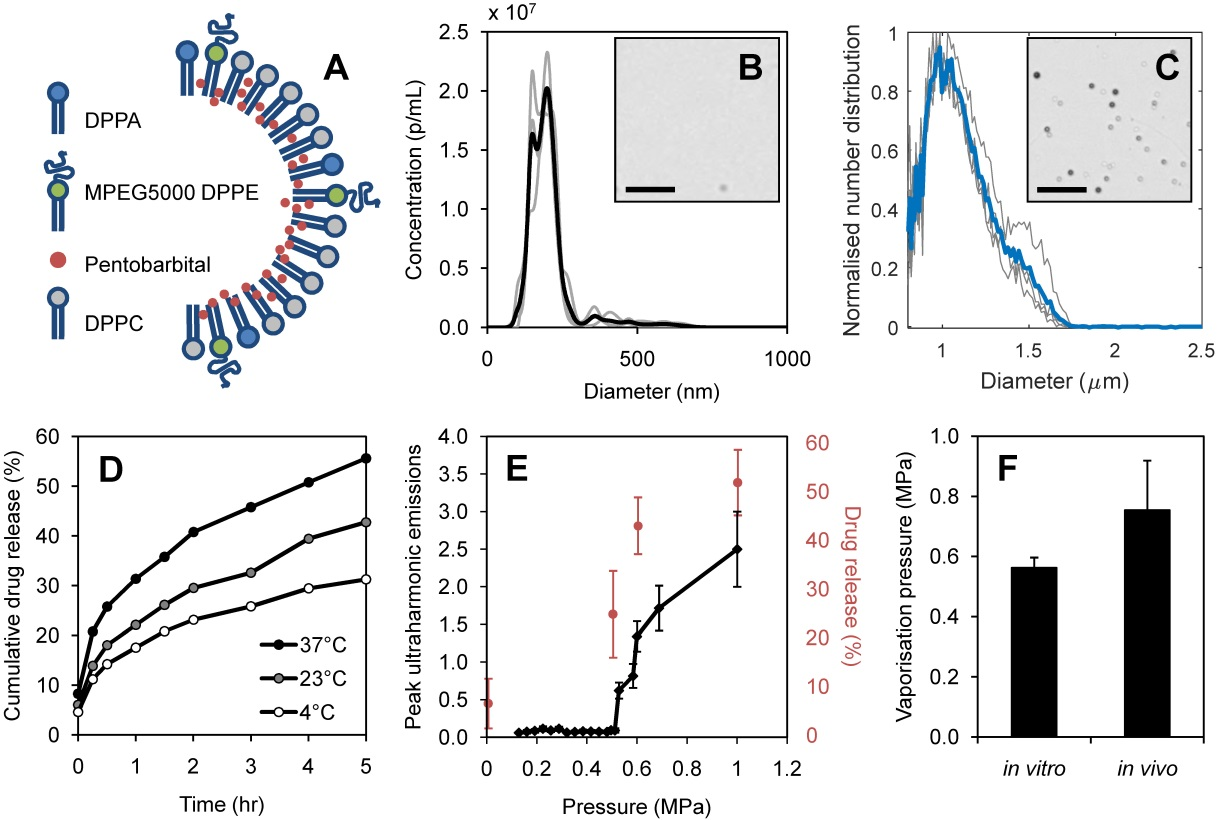
Nanodroplet characterization: **(A)** schematic of drug-loaded lipid shell composed of 1,2-dihexadecanoyl-sn-glycero-3-phosphate (DPPA), 1,2-dipalmitoryl-sn-glycero-3-phosphoethanolamine conjugated polyethylene glycol (MPEG5000 DPPE) and 1,2-dipalmitoyl-sn-glycero-3-phosphocholine (DPPC), **(B)** nanodroplet size distribution by nanoparticle tracking analysis at 1:10 dilution (n=3) with example brightfield image (scale bar 10 μm), **(C)** resultant microbubble size distribution measured by Coulter Counter (n=3) with example brightfield image (scale bar 10 μm), **(D)** spontaneous drug release measured in storage conditions of 4°C, 23°C, 37°C, **(E)** acoustic vaporization threshold *in vitro* shown through ultraharmonic emissions (black) alongside percentage of pentobarbital released (red) at increasing sonication pressures (n=6), **(F)** and comparison of vaporization pressures *in vitro* and *in vivo* (n=8), error bars show one standard deviation.

The vaporization threshold of nanodroplets *in vitro* was measured as 0.56 ± 0.03 MPa, showing a steep increase in ultraharmonic emission above this threshold (Figure 4E). A higher pressure was required to vaporize nanodroplets *in vivo*, giving a vaporization threshold of 0.76 ± 0.16 MPa estimated in the brain (Figure 4F). When exposed to pulsed focused ultrasound at pressures exceeding the vaporization threshold, substantial drug release was detected. Figure 4E shows the corresponding acoustic emissions and drug release as nanodroplets transform into microbubbles, for 3 independent batches. Non-specific drug release was found to be at 6.5± 5% of the total encapsulated pentobarbital volume. Beyond the vaporization threshold, increasing ultrasound pressure increased the quantity of drug released, reaching 51.9 ± 7% release efficiency at 1.0 MPa with 10 ms burst length (Figure 4E). The equivalent quantity of drug released spontaneously was measured after 5 hours of incubation at 37°C (Figure 4D).

### MRI-g-FUS

#### *In vivo* ultrasound-mediated pentobarbital delivery

**Figure 5:**
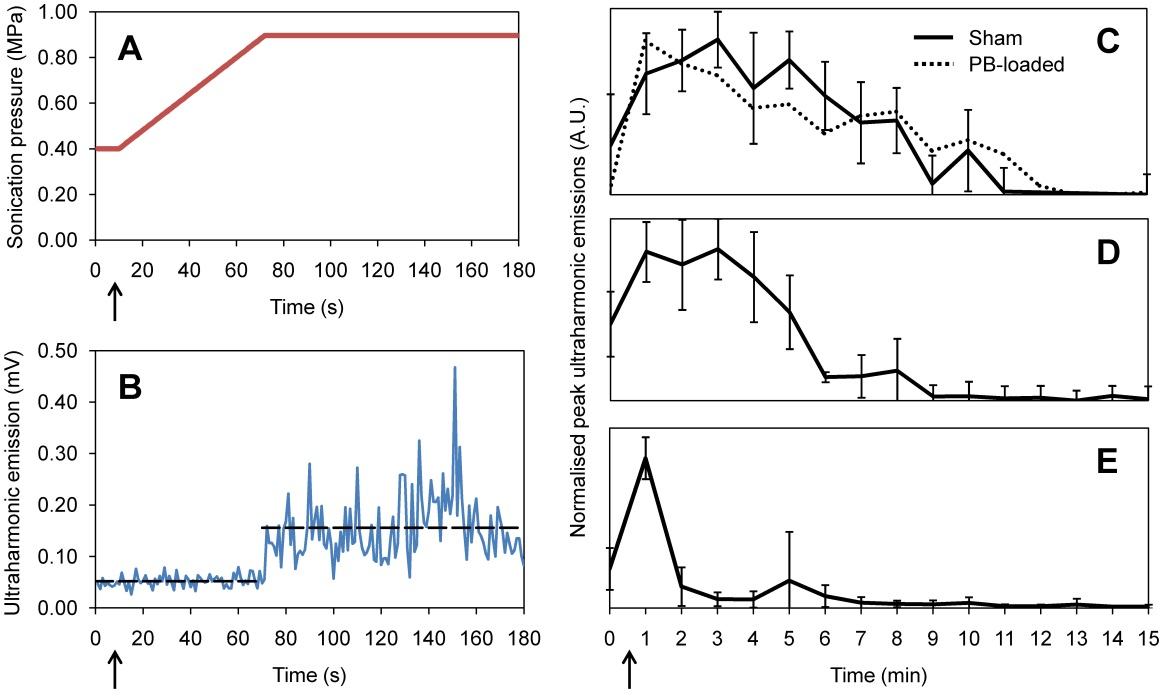
*In vivo* **(A)** feedback-controlled sonication pressure and **(B)** detected ultraharmonic emissions during a 180 s duration treatment, with droplet bolus injection at 10 s, indicated with arrows. Persistence of ultraharmonic emissions for sham and pentobarbital-loaded (PB-loaded) droplets in circulation *in vivo* at **(C)** 1 hour, **(D)** 3 hours and **(E)** 5 hours post-fabrication (time of nanodroplet bolus injection indicated with arrow). Each plot shows repetitions from 3 animals, each with an independent batch of droplets. Error bars show one standard deviation.

Figure 5A shows the ramping ultrasound pressure as dictated by the ultraharmonic control algorithm, reaching a plateau at the vaporization threshold pressure. As in *in vitro* studies, a jump in ultraharmonic emissions was seen beyond the vaporization threshold (Figure 5B).

The persistence of acoustic activity in circulation produced by the nanodroplets was found to be significantly greater than that reported for microbubbles, indicating a half-life *in vivo* of 8.4 ± 1.7 min, similar to that recorded in previous studies 9. Pentobarbital-loaded droplets showed a comparable half-life of 8.2 ± 0.5 min. Additionally, acoustic persistence was found to be dependent on the age of the nanodroplets. The nanodroplet half-life *in vivo* was measured as 8.4 ± 1.7 min, 5.2 ± 1.1 min and 1.7 ± 0.6 min after being stored on ice for 1 hour (Figure 5C), 3 hours (Figure 5D) and 5 hours (Figure 5E) post-fabrication respectively, where 3 animals were sonicated at each time point.

### Behavioral assessment

**Figure 6:**
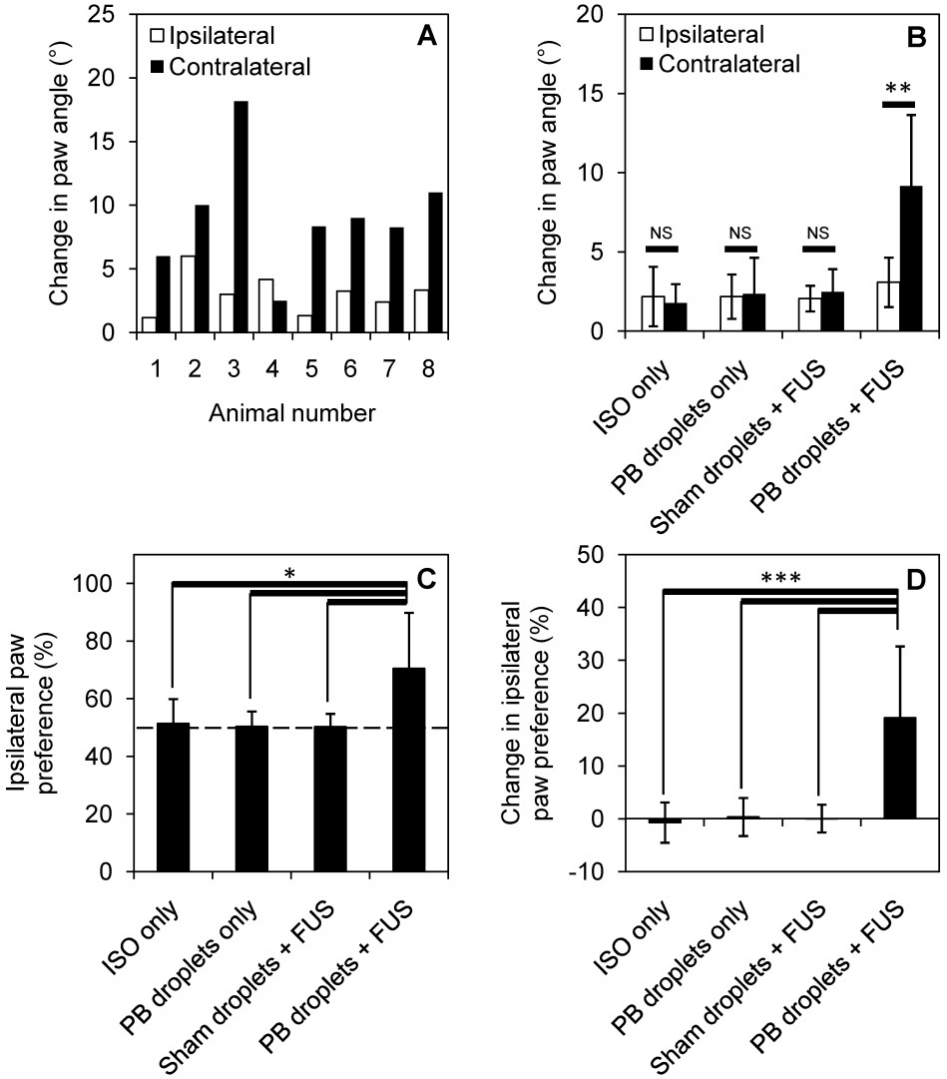
Behavioral changes induced by localized anesthesia in 8 treated animals and 5 animals in each control group. Change in paw angle is shown for **(A)** each treated animal individually and **(B)** summarized for each treatment group (**p<0.01, assessed by paired t-test). Ipsilateral paw preference is significantly increased for treated animals compared to control groups **(C) (D)**, error bars show one standard deviation and statistical significance is defined by p < 0.05 (*p<0.05, ***p<0.001, assessed by analysis of variance).

7 of 8 treated animals showed a greater change in paw angle on the contralateral side following treatment, indicating contralateral weakness through gait analysis (Figure 6A). When compared to control groups (ISO only, pentobarbital-loaded nanodroplets only, and sham droplets with FUS), pentobarbital-loaded nanodroplets with FUS showed a significant change in paw angle on the contralateral side (Figure 6B), assessed through a paired t-test (p < 0.05). 7 of 8 animals also showed an increase in ipsilateral paw preference following treatment, indicating ipsilateral forelimb dominance in the cylinder test (Figure 6B). When compared to control groups, pentobarbital-loaded nanodroplets with FUS showed a significant increase in ipsilateral paw preference (Figure 6C), assessed through a one-way ANOVA (p < 0.05). To account for any paw dominance prior to treatment, Figure 6D summarizes findings as change in ipsilateral paw preference (pre-treatment preference subtracted from post-treatment preference), showing 19 ± 13% change in paw preference in treated animals. 5 animals treated with pentobarbital-loaded nanodroplets and FUS were reassessed at 24 hours post treatment and all showed full behavioral recovery back to pre-treatment levels.

### Tissue assessment

**Figure 7:**
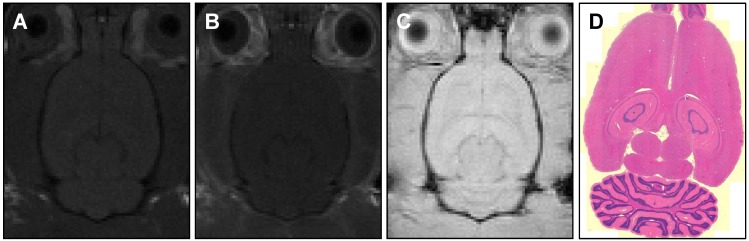
MR images of **(A)** pre-treatment (T1-weighted) and **(B, C)** post-treatment (T1-weighted, T2*-weighted) axial slices showing no detectable BBB-opening or damage. Histology sections **(D)** with H&E staining and 5 μm slice thickness also confirm no tissue damage.

**Figure 8:**
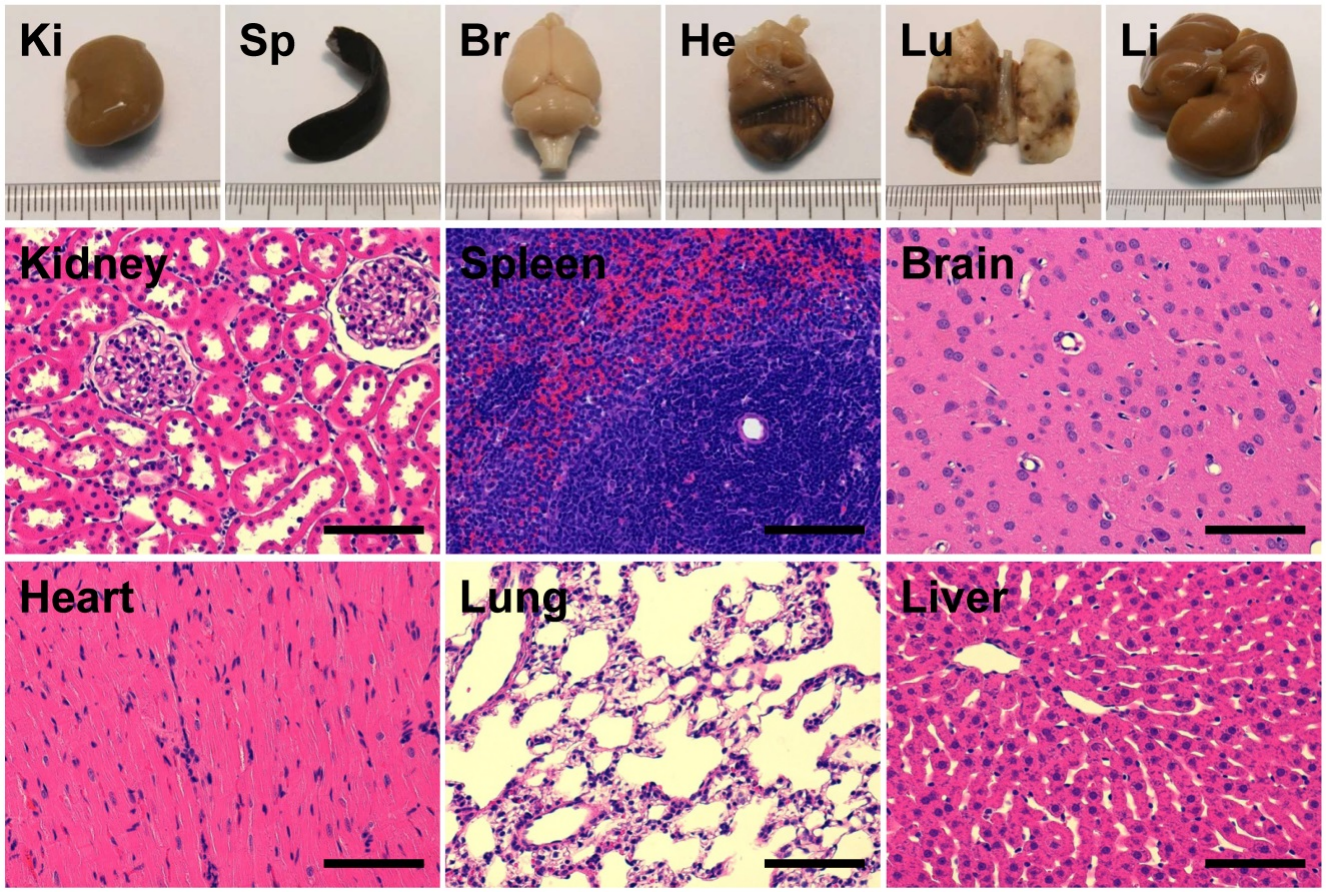
Assessment of gross histology and H&E stained sections from major organs - kidney (Ki), spleen (Sp), brain (Br), heart (He), lung (Lu) and liver (Li) - showed no signs of toxicity or necrosis (scale bars 100 μm).

Contrast-enhanced MR imaging showed no detectable BBB disruption or damage post-treatment (Figure 7A-C). Histological slides with H&E stain showed no observable red blood cell extravasation or detectable edema (Figure 7D, 7E). There were no signs of toxicity or necrosis in the gross histology or the H&E stained sections of kidney or liver (Figure 8). Histology also appeared normal in the heart, lung and spleen samples (Figure 8), suggesting systemic delivery of pentobarbital-loaded nanodroplets was well tolerated after 2 hours. For long term assessment, animals assessed for behavioral changes were monitored for multiple weeks following treatment and showed full recovery in motor ability and no signs of toxicity.

## Discussion

In this study we have shown for the first time that motor function of the rat brain can be temporarily inhibited using FUS to induce local, image-guided, release of an anesthetic from super-heated liquid nanodroplets. The nanodroplets were manufactured from a clinically-used commercial ultrasound contrast agent and vaporized using clinically-usable 580 kHz ultrasound. Therefore there could be a path for clinical translation using the approved clinical ablation device 22.

Focused ultrasound alone has been shown to modulate neuronal activity on a time scale of 10 - 40 minutes following sonication 23. In order to avoid measuring a transient effect from FUS alone, behavioral testing was carried out at 1 hour following sonication. The long acting barbiturate pentobarbital was chosen with an activity duration of 1 - 4 hours 24,25, allowing 24 hour post-treatment assessment of recovery. Furthermore, although pentobarbital has associations with toxicity and side-effects such as lethargy, incoordination, drowsiness and hypotension when delivered at high concentrations, it is still used clinically to induce medical comas in the context of traumatic brain injury. The current study uses a low concentration of pentobarbital, a quantity that only induces measurable effects when locally delivered 16. Pentobarbital is an attractive candidate for reducing systemic effects, targeting drug delivery and potentially inducing brain region-specific coma-like effects.

The biodistribution of lipid-based nanodroplets has been reported previously 26,27. Cao *et al.*^26^ shows predominant retention of nanodroplets in the liver after 24 hours, suggesting excretion through the bile. In fact, liver retention of nanodroplets is exploited by Phillips *et al.* 27 for enhancing ultrasound ablation, where an increase of droplets in the liver is seen after 95 min in a rat model. To illustrate that pentobarbital-loaded nanodroplets are well tolerated when delivered systemically, histology of major organ was performed which showed no signs of damage or necrosis after 2 hours. Furthermore, animals assessed for behavioral changes were monitored for multiple weeks following treatment and showed full recovery in motor ability and no signs of toxicity.

Instability and spontaneous vaporization of DFB nanodroplets after several hours has been reported previously 11 and is in agreement with our *in vivo* observations (Figure 5C-E). However, the lifespan of lipid-shell DFB nanodroplets can be extended by freezing at either -20°C or -80°C, then thawed at 4°C before use 28. Therefore we do not anticipate this 2 hour lifespan being a barrier to clinical translation.

Since pentobarbital is a small molecule drug (226 Da) that can pass through the intact BBB (as has been shown for other small molecule agents (<500 Da) and local anesthetics), local drug delivery was achieved even though the BBB was shown to remain intact after exposure to nanodroplets and FUS, indicated using contrast-enhanced MR imaging.

Interestingly, in previous studies nanodroplets of a similar lipid composition have been shown to disrupt the BBB, using sonications of a similar pressure (0.6 MPa) but at a higher frequency (1.5 MHz) 28,29. In contrast, polymer formulations used for drug delivery at frequencies closer to those used in the current study (0.65 MHz) detected no BBB disruption 2,6. Additionally, it has been shown that the presence of pentobarbital can prevent increases in BBB permeabilization in rats 30, although it is not known if that is the case with ultrasound induced BBB modulation.

However, the most likely reason for the BBB remaining intact following sonication is that of bubble size. In fabricating the nanodroplets we apply thorough size exclusion methods (triplicate washing and filtration) ensuring all erroneous large droplets are removed. We have verified experimentally - through microscopy and Coulter Counter measurements - that 99% of resultant bubbles are smaller than 1.61 μm in diameter. When compared to Wu *et al.* 28, where BBB disruption was recorded in a mouse model, the current study uses only 20.0% of the injected droplet volume (μL/kg) and 18.9% of the potential gas volume (μL/kg). Since it has been shown by Song *et al.* 31 that larger bubbles and greater injected gas volumes are more likely to disrupt the BBB, our small and tightly size-excluded resultant microbubble population reduces the probability of altering the BBB.

Although behavioral changes were indicative of successful drug delivery, future work will explore quantifying changes in neurotransmitters, in particular the increase in GABA expression and suppression of c-FOS associated with the delivery of pentobarbital. Similarly, more careful histology and behavior studies 32,33 are needed before clinical translation. Furthermore, the interval and safety of multiple exposures should be explored for possible repeated use of the method.

## Conclusions

In this study we have shown that a commercial ultrasound microbubble contrast agent can be loaded with a lipophilic drug and condensed to form drug-loaded nanodroplets. Ultrasound-triggered localized drug delivery to a specific brain region has been illustrated using a barbiturate anesthetic, at a sufficient local concentration as to induce a temporary contralateral motor deficit. MR images and histology showed no signs of BBB opening or tissue damage. The persistence of nanodroplet activity in circulation was measured up to 10 min following bolus injection, significantly longer than microbubble formulations, allowing a greater treatment window.

## Figures and Tables

**Table 1:** Number of animals distributed across experimental groups.

**Localized anesthesia study**	
ISO only	n = 5
PB droplets only	n = 5
Sham droplets + FUS	n = 5
PB droplets + FUS	n = 8
**Persistence study**	
PB droplets + FUS	n = 1
Sham droplets + FUS	n = 9
